# Electrospun Carbon Nanofibers from Biomass and Biomass Blends—Current Trends

**DOI:** 10.3390/polym13071071

**Published:** 2021-03-29

**Authors:** Imane Moulefera, Marah Trabelsi, Al Mamun, Lilia Sabantina

**Affiliations:** 1 Independent Researcher, Mostaganem 27000, Algeria; imanemoulefera@yahoo.fr; 2 Junior Research Group “Nanomaterials”, Faculty of Engineering and Mathematics, Bielefeld University of Applied Sciences, 33619 Bielefeld, Germany; marah.trabelsi@enis.tn (M.T.); al.mamun@fh-bielefeld.de (A.M.); 3 Ecole Nationale d’Ingénieurs de Sfax (ENIS), Department of Materials Engineering, Sfax 3038, Tunisia

**Keywords:** electrospinning, carbon nanofibers, biomass, biopolymers, energy storage, tissue engineering, CO_2_ capture

## Abstract

In recent years, ecological issues have led to the search for new green materials from biomass as precursors for producing carbon materials (CNFs). Such green materials are more attractive than traditional petroleum-based materials, which are environmentally harmful and non-biodegradable. Biomass could be ideal precursors for nanofibers since they stem from renewable sources and are low-cost. Recently, many authors have focused intensively on nanofibers’ production from biomass using microwave-assisted pyrolysis, hydrothermal treatment, ultrasonication method, but only a few on electrospinning methods. Moreover, still few studies deal with the production of electrospun carbon nanofibers from biomass. This review focuses on the new developments and trends of electrospun carbon nanofibers from biomass and aims to fill this research gap. The review is focusing on recollecting the most recent investigations about the preparation of carbon nanofiber from biomass and biopolymers as precursors using electrospinning as the manufacturing method, and the most important applications, such as energy storage that include fuel cells, electrochemical batteries and supercapacitors, as well as wastewater treatment, CO_2_ capture, and medicine.

## 1. Introduction

Increasing environmental awareness and ecological problems have led in recent years to new green materials made from biomass as precursors for the production of carbon materials (CNFs) receiving more research attention, as they seemed to be more attractive than traditional petroleum-based materials, which are polluting, toxic and non-biodegradable [1]. At the same time, there is a need to develop cleaner, more economical, efficient and energy-saving materials and to focus the world’s attention on the new green, renewable energies. Converting biomass waste into carbon materials can help solve the problem of pollution and improve traditional processing methods for producing carbon in the face of the energy crisis and environmental problems [1]. Low-cost renewable biomass materials, such as sawdust, wood residues, rice husks, and corn stover, among many others, are available in large quantities as waste from forestry and agriculture. These renewable biomass materials can be considered promising candidates for carbon precursors [2].

Various techniques for synthesizing carbon nanofibers from biomass, such as electrospinning, pyrolysis, hydrothermal treatment, and ultrasonic treatment, are already known and have already been explained by many research groups [3,4,5,6,7,8,9].

Most of the techniques are complicated and require extensive use of energy resources. For example, the production of carbon nanofibers from hazelnut shell biomass as a carbon resource by hydrothermal technique goes through a series of complex processes, such as hydrothermal carbonization, heat treatment, potassium hydroxide activation, magnesium oxide templating to produce anode materials for lithium-ion batteries at the end of the process [10].

This example shows the complexity of getting carbon nanofibers using traditional techniques, and therefore, the simple and low-cost production of carbon nanofibers by electrospinning offers an excellent solution. However, interestingly, only a few publications have been found on obtaining carbon nanofibers from biomass using the electrospinning method. It seems likely that other methods have been more researched than electrospinning, which has seen a rapid increase in publications in this area in recent years. In addition, the lack of research could be because more well-known polymers are used for the production of nanofiber mats, and the field of biomass is slowly making its entry into electrospinning.

Moreover, the biomass forms a complex composition of different substances that cannot be electrospun alone and should be extracted first, furthermore requiring polymers, such as PAN or PEO, as a carrier material to stimulate nanofiber spinning. The considerations at this point can be diverse, and this review attempts to fill this gap.

Electrospinning is a well-known process for producing nanofibers from various synthetic and bio-based polymers and is very easy to apply [2,11]. Carbon nanofibers can be obtained from nanofibers produced by electrospinning technology and subsequently thermally processed. Nanofiber mats result from producing fibers with diameters of a few tens to a few hundreds of nanometers from a polymer solution or melt in a strong electric field [12,13]. The areas of application for such nanofiber mats are broad due to the numerous positive properties, such as large surface-to-volume ratio and outstanding flexibility, etc. The compositions of electrospun fibers are diverse and much more exciting than any other fibers. Electrospun nanofibers can be made from almost unlimited materials, from synthetic to natural polymers, and by adding particles, new materials and composites can be produced [14,15,16,17]. Biomass-derived carbon nanofibers can be produced from a variety of polymers, such as chitin from seafood [18] or fungi [19], lignocellulosic materials [20], gelatin, proteins and from the combination of polymer blends. In order to manipulate the bonding between the fibers of the nanofiber material and improve the overall electrical conductivity, mesoporosity and surface area, often (polyacrylonitrile) PAN-based carbonized nanofibers are used, blended with biopolymers because producing nanofibers from these biopolymers alone is not possible. Nanofibers can be produced from these bio-based polymer blends at a much lower cost than the conventional processes currently used [21]. These bio-based polymer blends are used for various commercial applications in the industrial field and are specially developed for this purpose. Such precursor electrospun fibers and carbon fibers are of great interest for a range of applications, including drug delivery and regenerative medicine [22], tissue engineering [23], cells growing [24], catalysts [25], filters [26,27,28] or protective clothing [29]. Not only artificial polymers [30] and biopolymers [31,32] can be used for the production of nanofiber mats, but also polymers mixed with non-soluble materials can be used for electrospinning [33,34]. By adding particles, it is also possible to produce magnetic nanofiber mats for various applications [35,36,37]. These magnetic nanofibers are promising for basic research and future applications in spintronics or neuromorphic computing [38,39,40,41,42]. Due to their high surface-to-volume ratio, nanofibers are ideal for filtration applications [43]. As a result of their high porosity and permeability, as well as their small pore size, reliable filtration of the smallest and finest particles can be achieved [44,45]. Other applications of nanofiber mats as filters include optical and chemical sensors [46], energy storage [47], aerospace [48], nanocatalysis [49], protective clothing [50], transportation [51], water and air filters for biotechnological and industrial applications [52,53,54,55,56,57,58], filtering of dye particles from wastewater from the finishing industry [59] or filtering of smoke filtration and flavor retention [60]. Great research interest in recent years shows the use of biomass for the production of nanofibers from the biomass and natural biopolymers used as a precursor for carbon and carbon nanofibers to make the contribution to the ecological point of view and the extraction of raw materials from natural resources. This overview summarizes and presents the current developments. Furthermore, the research gap in the production of carbon nanofibers from biomass by the electrospinning method will be discussed. Carbon nanofibers derived from biomass provide an alternative method for various industrial applications, reducing economic costs and environmental impact.

## 2. Biomass as Precursor for Carbon Nanofibers

Various natural substances have been used as precursors for the synthesis of carbon materials, such as activated carbon and carbon nanofiber [61]. Biomass is any organic matter that is available on a renewable or recurring basis. It can be classified into forest residues, agricultural residues, food industry waste, municipal solid waste, industrial waste, wastewater, and animal waste [4]. In recent years, the use of biomass as a precursor for carbon and carbon nanofiber has been widely explored due to their contribution to the ecologic system [62] and also due to the low cost of the extraction of the raw materials. Various biomasses used for the production of carbon nanofibers include lignocellulose materials [63,64,65,66,67,68,69] such as cellulose, hemicellulose [70], lignin [71,72], bamboo [73], crab shell [74], natural fungus [75,76], sawdust [9], seafood chitin [77], coconut shell charcoal [78] etc. On other hand, carbon nanofiber also can be prepared from polymers, such as polyacrylonitrile (PAN) [79,80], poly(vinyl alcohol) [81], vanillin polymer [82] and poly-(vinyl pyrrolidone) [83].

## 3. Preparation of Carbon Nanofibers

### 3.1. Electrospinning

Electrospinning is a well-known simple, productive process for producing highly functional fibers in the micro- and nanometer range [84]. The advantages of nanofibers include easy and low-cost manufacturing, lightweight, high molecular orientation, and high flexibility in surface functionality, as well as almost unlimited use of biobased and synthetic polymers and blends of various particles [85]. In all electrospinning techniques, such as needle-based, needle-free, electrospraying and others, a high-voltage electric field is basically applied to a polymer solution, causing the fibers to form a Taylor cone at the tip [86].

**Figure 1:**
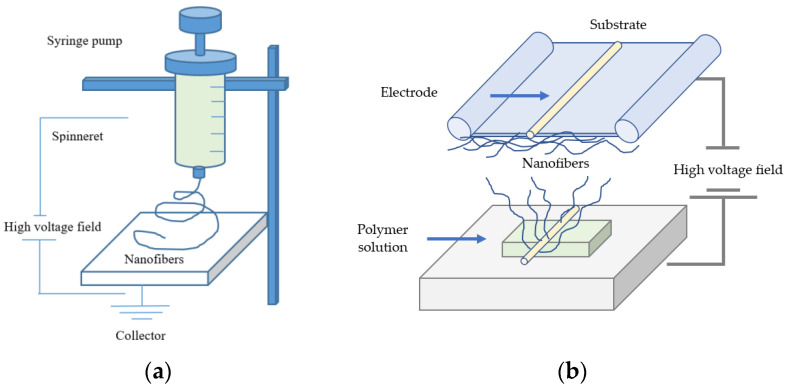
(**a**) A basic setup of needle-based and (**b**) needleless electrospinning methods.

A strong electric field carries electric charges along the polymer surface, and Taylor cones are formed. Subsequently, the droplets of electrospinning solution are attracted to the counter electrode. As a result, the droplets are stretched extensively and form a very thin nanoscale fiber when they reach the substrate [87,88]. In the electrospinning process, a polymer solution or melt is generally deposited through a needle [89,90] or in needleless technique by coating a wire, cylinders, other objects that are used as electrodes or free surfaces with the polymer melt or solution [91,92,93]. The basic setup of electrospinning involves the application of a strong electric field to a droplet of the polymeric solution used (e.g., precursor melting solution). The typical setup of electrospinning essentially consists of a high-voltage field, a syringe pump, a spinneret, and a collector onto, which the nanofibers are deposited [94]. A basic setup of needle-based and needleless electrospinning principles is shown in Figure 1.

The electrospinning parameters, such as voltage, feed rate, electrode-type, and distance between electrode and collector, significantly affect the properties of the resulting electrospun nanofibers [95]. The electrospinning parameters play a decisive role in the resulting nanofibers. Depending on the change of the parameters, the defined nanofibers with desired properties can be produced [96,97].

### 3.2. Stabilization and Carbonization of Nanofibers

**Figure 2:**
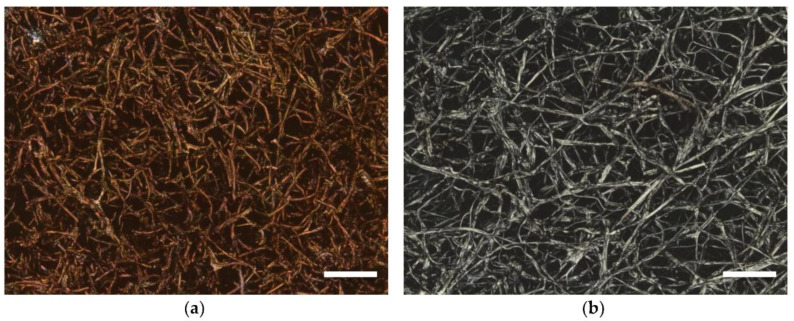
Confocal laser scanning microscopy (CLSM) images of oyster mushroom mycelium on polyacrylonitrile (PAN) nanofiber mats (the latter not visible) (**a**) after stabilization of the whole composite for 1 h at 280 °C; (**b**) after carbonization for 1 h at 500 °C of the whole composite. Scale bars indicate 20 µm. Reproduced from [19], published under a CC BY 4.0 license (MDPI, Basel, Switzerland).

Various biobased and synthetic polymers are used as precursors for the preparation of CNFs by electrospinning followed by thermal heat treatment process involves oxidative stabilization and subsequent carbonization for the production of carbon fibers [98,99]. Oxidative stabilization is used because it prevents melting or fusing of the fibers and thus increases the final elemental carbon content. In other words, the stabilization process minimizes the volatilization of elemental carbon to maximize the final carbon yield [100]. Both thermal processes exert a great influence on the fiber morphology and the resulting properties [101,102]. Figure 2 depicts the carbonized mycelium/PAN nanofiber composite.

**Figure 3:**
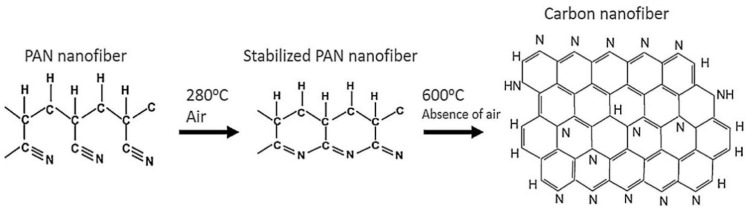
The effect of thermal treatment in PAN structure change (proposed by Ibupoto et al.) Reprinted with permission from [105]. Copyright (2018) by Elsevier Publishing.

The dimensional change of the nanofiber mats takes place when the temperature is increased during the stabilization process. As the temperature increases, the dimensions of the samples will decrease. These changes happen on the molecular level due to the prevailing processes during the isothermal treatment. During the stabilization process in air, various processes, such as cyclization, dehydrogenation, oxidation, aromatization, and crosslinking reactions, take place, leading to an aromatic ladder structure [103,104]. These processes reduce the size of the sample area and the weight of the sample. The stabilization process plays an important role in the properties of the resulting CNFs and should be carried out in a controlled way. After stabilization, carbonization is performed, which is usually carried out in a nitrogen atmosphere and leads to different chemical and morphological properties of the carbon nanofiber depending on the treatment temperature. Figure 3 shows the changes at the molecular level and the transformation of PAN nanofiber mats into carbon nanofibers.

The carbonization process typically occurs under inert gases, such as argon or nitrogen, at temperatures ranging from 500 °C to 1.200 °C (see Table 1). The morphological modification of the resulting CNFs can be achieved by using different techniques. The polymers can be completely removed from the fibers during thermal treatment by pyrolysis [106,107] or by adding ingredients, such as phosphorus [108], nitrogen [109] or particles [110] to modify electrical or mechanical properties. By using blends, carbon nanofibers with adjustable fiber morphology can be produced.

During the thermal exposure, not only the dimension of nanofiber mats but also their color changes significantly. After electrospinning, the nanofibers have a matte white color, which changes with the increase of temperature from white to yellow, golden brown and at about 280 °C to dark brown. With the increase of temperature during the carbonization process, the CNFs have a black color. Moreover, after the electrospinning process, the nanofiber mates prove to be very flexible and with the increase of temperature, they become more unstable and brittle, and CNFs break very easily.

Table 1 summarizes some different carbon nanofibers obtained from biomass and biopolymers or a mixture between synthetic and biopolymers prepared by electrospinning.

As shown in Table 1, carbon nanofibers are largely produced from lignin and cellulose-containing biomass. Carbon nanofibers prepared from PAN/fungal mycelium or PAN/gelatine are still a novelty. Lignin and cellulosic materials have been known for a long time and have been intensively researched, and therefore, several studies are available, which deal with these materials.

## 4. Application of Carbon Nanofibers

**Figure 4:**
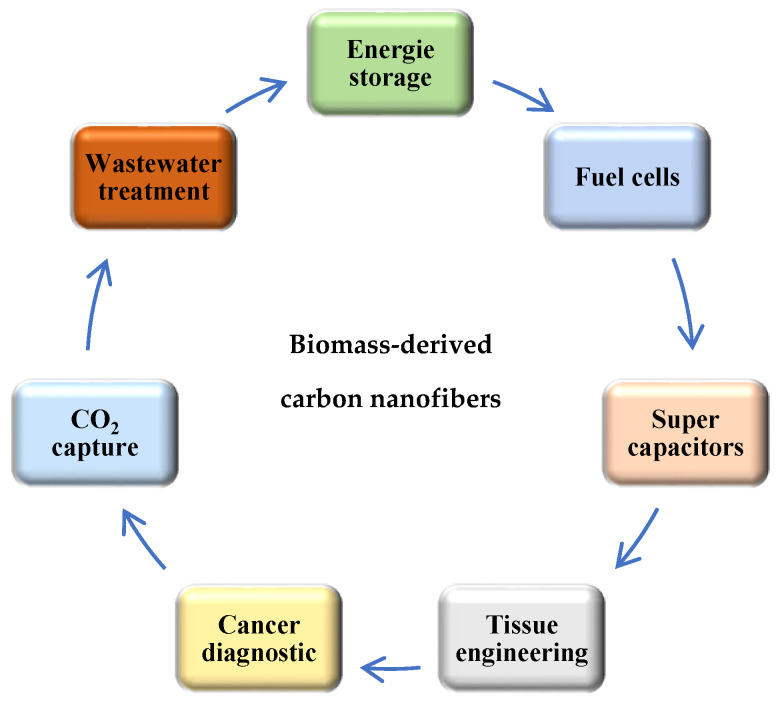
The different applications of biomass-derived carbon nanofibers.

Electrospinning is one of the most productive methods for the preparation of carbon nanofiber using different precursor solutions. Recently, many authors have intensively studied the production of carbon nanofibers by electrospinning from biomass and polymers with or without additives, such as various metals, particles, acids, alkyls, to increase the importance of their application in industry. The importance of carbon nanofibers is based on their porous structure, high specific surface area, high conductivity, and thermal stability. All these properties make the development of the preparation methods a big challenger for recent works. Recently, the manufacturing of carbon nanofibers has significantly improved diverse applications due to their physical and chemical properties, such as high porosity and large specific surface area, numerous active sites, good catalytic properties, high conductivity, good temperature stability, and low-cost, which have been a challenge for many industrial applications (Figure 4), such as energy storage (fuel cells, electrochemical batteries and supercapacitors) [120,121,122], environment science [123,124], tissue engineering [125], optical sensors [126], cancer diagnosis [127].

### 4.1. For Energy Storage

#### 4.1.1. Fuel Cells

Fuel cells function as batteries, which are electrochemical cells applied to convert chemical energy into electricity through two electrodes (cathode–anode) by redox reactions. The application of rechargeable ion batteries and polymer electrolyte membrane fuel cells has aroused interest in analytical models for calculating the transverse permeability of the gas diffusion layer in proton-exchange membrane fuel cells [128] and effective electrolyte diffusivity considering the electrokinetic effects and microstructure parameters of porous media using the using the fractal theory of porous media [129]. Fuel, such as hydrogen, is supplied to the anode, and the air is supplied to the cathode. Presently, most commercial catalysts used for fuel cells are catalysts doped with metal as Pt due to their high efficacity and stability. However, the high cost of this metal decreases the utilization of this type of fuel cell.

Recently many investigations used carbon nanofiber for fuel cells due to their large surface area, higher conductivity, and stability. Among them, Chung et al. used CNFs prepared by electrospinning of PAN following by heat treatment of stabilization, carbonization and graphitization at different temperatures for Pt/C cathode to improve water management in fuel cells [79]. They concluded that the enhanced air transport in the water-free region in the cathode provided by hydrophobic carbon glass fibers CGFs also suggested this principle for an effective water management method in the electrode. Abdelkarim et al. summarized that Ni-decorated carbon nanofibers from poly(vinyl alcohol) doped with Cd could be an effective co-catalyst for urea oxidation comparable with Ni-carbon nanofibers [81]; they also found that the quality of electrochemical oxidation activity and the number of active sites were improved. In another work, Chan et al. analyzed the performance of carbon nanofiber for polymer electrolyte membranes fuel cell catalyst layers [130]. The results showed that the carbon nanofiber-based catalyst layers significantly outperformed traditional carbon-supporting catalyst layer structures. Also, further optimization can be achieved by improving the control of the morphology and distribution of ionomers, especially in terms of ionomer bridges and connectivity. On the other hand, Ponomarev et al. prepared a complex carbon nanofiber paper by electrospinning PAN with integrated Zr and Ni, followed by further pyrolysis to use it for the high-temperature (HT)- proton exchange membrane fuel cell (HT-PEMFC) [131]. They confirmed that the use of the carbon nanofiber platinum support obtained leads to a higher fuel cell efficiency comparable to the Celtec^®^ P1000, which is one of the best commercial electrodes.

#### 4.1.2. Electrochemical Batteries

Carbon nanofibers can show excellent electrical conductivity, leading to their use as a leading additive in lithium-ion (LIB) and sodium-ion (NIB) electrodes. In the last years, several authors investigated the use of carbon nanofibers in batteries as a novel approach. Amongst them, He et al. reported that TiO_2_/carbon nanofibers obtained from PAN by electrospinning showed high performance as negative electrodes for vanadium redox flow battery (VRFB) [80]. The authors observed that the cell using CNF–TiO_2_ as a negative electrode showed an increase of 7.8% in the energy efficiency than the pristine cell. Also, Trung Bui et al. synthesized carbon nanofibers from PAN and PVP (polyvinyl pyrrolidone l with metal (Pt, Co, and Pd) by coaxial electrospinning to improve the performance of Li–O_2_ battery cathodes [132]. The results indicated that the use of CNF–Pt in Li–O_2_ cells displays much better electrochemical performance in specific capacity, rate capability, energy efficiency, cycle stability, and O_2_ efficiency, comparable with the two other metal-embedded carbon nanofibers (CNF–Pd, CNF–Co). They also observed a reduction in overpotential in both charging and discharging with an improvement in cycle life with a limiting capacity of 1000 mAh/gc at a current density of 500 mA/gc. Furthermore, due to the importance of improving the performance of Li–O_2_ battery cathode, Yoon et al. evaluated the use of carbon nanofibers with metal using cobalt nitride (Co_4_N) [133]. The authors found that the cathode (Co_4_N/CNF) had excellent electrochemical performance and exceptional stability over 177 cycles in Li–O_2_ cells.

The results proved that the favorable formation/decomposition of reaction products and the transmission of side reactions are largely regulated by the appropriate surface chemistry and adapted structure of the cathode materials, which are necessary for lithium batteries. Furthermore, Xia et al. investigated the use of flexible SnSe/C nanofiber membranes by simple electrospinning, followed by calcination, in both LIBs and SIBs batteries [134]. The findings obtained prove the high-performance of the capacity and cycling stability in the batteries. In addition, LIBs battery displayed a higher discharge capacity at higher current density after 500 cycles and the same for SIBs, but after 200 cycles at different current densities.

On the other hand, carbon nanofibers are also used for sodium-ion batteries. Zhao et al. manufactured carbon nanofibers from PAN doped with NiS_2_ nanoparticles by electrospinning [135]. The authors reported that NiS_2_ nanoparticles/carbon nanofiber as SIBs anode display a high-performance with a good specific capacity of 500 mAh g^−1^ at 0.1 A g^−1^ and also a competitive rate with storage of 200 mAh g^−1^ at 2 A g^−1^, in addition to long-lasting consistency within 1000 cycles. Besides, Zhou et al. studied the performance of the ZnSe electrospinning nanofiber form PAN in lithium and sodium batteries as anode materials [136]. They showed that ZnSe@N–CNFs present an excellent performance in LIBs and also in SIBs at higher current density. Furthermore, the sample exhibited a high capacity for both batteries (LIBS and SIBs).

Yin et al. prepared CoSe_2_ grain attached carbon nanofiber from PAN as a carbon source by electrospinning for flexible sodium-ion batteries [137]. It was demonstrated that the carbon nanofibers form a flexible, binder-free film and thus a three-dimensional, conductive, interconnected network. Due to the carbon nanofiber structure, it was confirmed that electrochemical conductivity and Ni ion diffusion, structural stability and also cycling stability were improved.

#### 4.1.3. Supercapacitors

Supercapacitors are a kind of new energy storage and conversion equipment that should have the potential for high-power density, high circulation characteristics, fast discharge/charge, low self-discharge, safe work, and low cost. Various porous materials, including porous carbon and carbon nanofibers, are used in the manufacture of supercapacitors due to their high electrochemical properties, such as resistance, conductivity, and good temperature stability, also due to their high specific surface area and porosity. Currently, many investigations have been carried out on the use of carbon nanofibers obtained from electrospinning as supercapacitors.

Dai et al. summarized the use of lignin/PAN with graphene doped with nitrogen-sulfur carbon nanofiber obtained by electrospinning for supercapacitor. They mentioned that the high surface area obtained of 2439 m^2^ g^−1^ and good surface morphology of the carbon nanofiber exhibited a supercapacitor with high energy and high-power densities, also ultra-high capacity with cycling stability of 97.8% after 5000 cycles of charge/discharge [138] Cao et al. also tested cellulose acetate/lignin-based carbon nanofibers as supercapacitors [72]. The cellulose/lignin-based carbon nanofibers showed a high energy density of 30.2 Wh/kg at a power density of 400 W/kg due to the covalent bond of lignin and cellulose acetate, which give the material good thermal stability, high specific surface area, filamentous morphology, homogenous diameter distribution and high storage capacity. Besides, Ma et al. evaluated carbon nanofibers from lignin and PVP by electrospinning with Mg(NO_3_)2·6H_2_O as activate agent at different ratios as an electrode in supercapacitor [112]. The authors suggested that the increase in the ratio of Mg(NO_3_)2·6H_2_O increases the surface area and the mesoporosity of the carbon nanofiber, leading to an improvement in capacity when it used as triple- and double-electrode, also they observed that lignocellulose carbon nanofibers (LCNFs) present a good outstanding rate performance and high cycling stability. On other hand, García-Mateos et al. synthesized carbon nanofiber from lignin with phosphoric acid by electrospinning following by two separate heat treatments in air and in an inert atmosphere to be used in supercapacitor electrodes [66]. They reported that the activation under air atmosphere increases the energy storage up to 50% and also ameliorated the power capability of the electrode. In the study by Wang et al., carbon was fabricated from biomass, with a large number of straight holes embedded with nickel particles as the anode framework. The nickel-modified electrode was stably cycled for 1370 h at a current density of 5 mA/cm^2^ with a capacity of 2 mAh/cm^2^, and lithium storage with a more substantial capacity (5 mAh/cm^2^) was used, which kept the cycling stable for 630 h. Furthermore, the nickel-modified electrode was used as the anode. Biomass carbon matrix with pores and sulfur powder was filled and tested as the positive electrode. According to the study, the capacity retention rate is 85% after 100 periods of full cell operation [139].

### 4.2. Environmental Science

Carbon materials, such as activated carbon and carbon fibers, are widely used in environmental science, more specifically in adsorption and absorption and liquid or gas phases. Adsorption is a phase transfer process mostly used in purification and separation processes and, particularly, in the removal of pollutants from the fluid phase [140]. The process of absorption means that material catches and transforms energy. Therefore, absorption distributes content that it catches through the whole bulk, while adsorption distributes it only along the surface.

#### 4.2.1. Wastewater Treatment

The removal of pollution from wastewater has become a significant challenge for many investigations due to their impact on human health, the environment, and aquatic environments [141]. Nowadays, carbon nanofibers adsorption is one of the most popular technologies for wastewater treatment [142] due to their physical and chemical properties, such as high specific surface area, porosity, surface chemistry and morphology.

Zhao et al. evaluated the use of nanofiber membranes made of biomass vanillin polymer for the adsorption of methylene orange and sodium dodecyl sulfate [82]. They reported that the adsorption capacities reached their maximum at 406.6 and 636.0 mg/g for MO and SDS, respectively. Furthermore, they mentioned that the regeneration of biomass vanillin-derived polymer nanofiber has a high efficiency even after six adsorption-desorption cycles. Recently, Widiyastuti et al. analyzed electrospun activated carbon nanofibers from coconut shell charcoal and PVA with KOH for the adsorption of methylene blue [78]. They concluded that the activated carbon nanofiber obtained at PVA 12 *w*/*v*%, 60 wt % C displays the highest adsorption capacity of 166.7 mg/g with the ability to remove 96% at the third cycle.

In addition, some authors carried out the use of the photocatalytic process for wastewater treatment. Among them, Gan et al. analyzed the preparation of biomass cellulose-derived carbon nanofibers by electrospinning with bismuth oxybromide (BiOBr) at different pyrolysis temperatures for the removal of rhodamine B (RhB) and hexavalent chromium (Cr(VI)) [143]. They summarized that the increase in pyrolysis temperatures leads to an increase of the photocatalytic capacity for both RhB and Cr(VI). Additionally, they confirmed that the BiOBr/cellulose-derived carbon nanofibers CCNFs show a high efficacity for the simultaneous removal of the contaminants (RhB and Cr(VI)).

#### 4.2.2. CO_2_ Capture

A large number of greenhouse gas emissions currently contribute to global warming and pose a worrying threat to the environment and human health [142]. Among all greenhouse gases, CO_2_ comes from many sources and has a significant impact on global climate change [142]. Actually, many technologies are used for the capture of CO_2_, the most often applied, of which are absorption, adsorption, membranes, cryogenic, enzymatic and hybrid.

Adsorption is the most commonly used method due to its effectiveness, selectivity, renderability and cost-efficiency. Carbon nanofibers are considered as one of the best alternatives for CO_2_ capture because they have not only the intrinsic properties of carbon nanomaterials but also an adjustable pore structure [113,142].

In 2016, Calvo-Muñoz et al. successfully manufactured carbon fibers from biomass by electrospinning, where they used alcell lignin with ethanol as precursor fuel cell letter (FCL) for CO_2_ capture under post-combustion conditions [113]. The results revealed that FCL exhibited capacity values of 1.3 mmol/g, with full regeneration of the carbon material at the same condition without any additional energy. More recently, Zainab et al. analyzed the efficient CO_2_ capture by polyacrylonitrile–poly(vinyl pyrrolidone) (PAN/PVP) carbon nanofibers obtained via electrospinning with subsequent intensive washing and carbonization treatment [83]. The authors suggested that the PAN/PVP carbon nanofibers showed a high selectivity with a CO_2_ adsorption capacity of 3.11 mmol/g. They also mentioned the high selectivity of the regeneration up to 50 cycles of adsorption/desorption.

Ali et al. tested polyacrylonitrile–polyvinylidene fluoride (PAN/PVDF) core-shell nanofibers hybridized with tin oxide nanoparticles for adsorption of CO_2_ [144]. The maximum capacity obtained was 2.6 mmol/g at room temperature, where they confirmed that the carbon nanofiber displays high remarkable cycling stability even after 20 adsorption/desorption cycles and reached a maximum of 95% of the initial CO_2_ adsorption capacity. Furthermore, Chiang et al. also prepared nanofiber adsorbents from polyacrylonitrile (PAN) via electrospinning, following by different heat treatments (stabilization, carbonization, and activation) for CO_2_ adsorption application [145]. They reported an adsorption capacity of 1.2 mmol/g (0.15 atm) and 3.2 mmol/g (1 atm), obtained at 298 K, and explained the high efficiency of the carbon nanofibers by the activation temperatures

### 4.3. Biotechnological and Medical Fields

Today’s medicine progresses greatly and applies more therapeutic solutions based on the field of nanotechnology and nanomaterials. High-performance materials, such as carbon nanotubes, graphene, or carbon nanofibers, have already established their place in developing new implants and medical devices [141]. Due to their properties, high electrical conductivity, unique surface characteristics, and biomimetic shape, these nanomaterials are ideal for constructing implantable electrodes and biosensors. In addition, they can serve as tissue substrates for in vitro and in vivo applications. For this reason, stimulation of an electric field can regulate cell behavior both in vivo and in vitro due to the conductive properties of carbon substrates. Nanofibers resemble the natural structure of cell assembly and can be used in the form of porous mats as membranes for medical reconstruction, substrates for bone and cartilage development in post-traumatic tissues [146,147].

Polymer nanofibers, as well as carbon nanofibers, are promising candidates for diverse medical applications thanks to their physical properties. Due to their conductivity, they can be used as biosensors and electrodes to stimulate the nervous system, as well as for the fabrication of scaffolds for regenerative medicine. As nonwovens, mats, membranes, or other various types of nanocomposites, nanofibers can be used in many biotechnological fields [148,149,150,151,152]. Aoki et al. investigated the application potential of organic nanofibers and electrospun carbon nanofibers for bone regenerative medicine [153].

Previously, the research focus centered on coating nanofiber mates with antibacterial substances. The efficacy of silver nanoparticles and the active healing properties of chitosan polymer hydrogels received numerous publications. With the development of electrospinning processes, the research focus increasingly shifted to electrospun nanofibers, which exhibit antimicrobial properties through the addition of nanoparticles [154,155]. Due to their high mechanical strength and good biocompatibility, carbon-based nanofibers offer further areas of application in biomedicine [156,157,158].

In 2019 Li et al. conducted a study of a superhydrophobic hemostatic material made from a nanocomposite dispersion of a dense network of carbon nanofibers and polytetrafluoroethylene (PTFE) or poly-dimethylsiloxane (PDMS) on support [159]. This nanofiber material has been used for its particular and distinctive way of blood coagulation, which allows rapid blood coagulation due to the presence of microfibers and reduces subsequent blood loss, regardless of the pressure applied, due to its superhydrophobic characteristics.

In tissue engineering for regeneration or organ reconstitution, cells are designed to attach, proliferate, multiply and regenerate multiple organs, such as skin, bone, cartilage, muscle, tendons, heart, nerves, and blood vessels. These strategies depend on appropriate biochemical and physicochemical properties and molecular influences or control of cellular behavior [160]. Carbon nanofibers are potential candidates for tissue engineering applications because they have suitable physical, structural, mechanical, and biological properties [161,162,163]. In addition, carbon nanofibers have exceptional mechanical properties, conductivity, and excellent cytocompatibility properties, as well as osteoblast adhesion, which are suitable for neural and bone tissue engineering applications. In terms of carbon nanofiber adhesion and proliferation, they show the interaction of astrocytes like glial scar tissue-forming cells. These functions of astrocytes make them able to minimize nanoscale fibers and scar tissue formation, reduce the glial scar tissue formation and show positive interaction with neurons, which would be a great support for neural implants [164,165,166].

Recent research indicates that carbon-based nanomaterials are potential candidates for biomedical applications, including drug delivery, repair and regeneration of various tissues, including nerves, muscles, bones, and for imaging [167,168,169]. Stocco et al. have investigated that carbon nanofibers have strong mechanical properties capable of surviving without affecting mesenchymal stem cells for tissue engineering of the knee meniscus [170]. Samadian et al. and Patel et al. have found that carbon nanofibers are promising platforms with a nanoscale surface area that are helpful for tissue healing and bone regeneration process through anti-inflammation, pro-angiogenesis and stem cell stimulation [169,171]. The research group of Serafin et al. has presented that the electrically conductive properties of carbon nanofibers can be used in cardiac or neural tissue engineering applications [172]. In addition, carbon nanofiber composites have special properties, such as large specific area, high porosity, good biodegradability, cytocompatibility and conductivity, etc., making them ideal candidates in the field of tissue engineering and biological medicine [173,174,175,176].

In addition, there is a wide range of further carbon nanostructures such as carbon nanotubes, carbon nanofibers, carbon nano-onions (CNOs), graphene, which have attracted a lot of attention recently due to the promising industrial application areas. Onion-like quasi-spherical CNPs (OCNPs) with hollow cage-like concentric graphene shells have been known since 1992 but are still under-researched compared to other allotropic forms of nanocarbons, such as carbon nanotubes, carbon fibers, fullerenes, graphene, and carbon dots. CNOs are a niche product that has not been explored as much as other carbon nanostructures and offer many advantages, unlike other carbon nanostructures. They exhibit lower toxicity, have one of the exceptional biocompatibilities and are, therefore, of particular interest for medical and biotechnological applications, such as imaging, drug delivery, tissue engineering, sensing and as [177,178,179]. Excellent electrochemical performance is offered by CNOs due to their high surface area, the small size of the carbon-oxygen functional groups and the micro-open 3D graphite structures. These properties provide sufficient space for ion storage, hierarchical porous channels for ion transfer and a carbon matrix with high conductivity for electron transfer [180]. Breczko et al. prepared composites of CNOs and poly(diallyldimethylammonium chloride) (PDDA) or chitosan (chit), and the electrochemical properties were tested and investigated [181]. In another study, CNO–PDDA composite films for dopamine detection were prepared in the presence of ascorbic acid and uric acid in solution [182]. The research group of Giordani et al. prepared a novel near-infrared (NIR)-fluorescent carbon-based nanomaterial, which consists of boron-difluoride azadipyrromethene fluorophores covalently bonded to carbon nano-onions [183]. The cytotoxicity and immunomodulatory properties of the synthesized fluorescein CNO derivative were elucidated and compared with similarly functionalized CNTs. CNOs were found to exhibit efficient cellular uptake, mild inflammatory potential, and low cytotoxicity. These discoveries make CNOs promising materials for biomedical application areas. Moreover, due to a novel concentric graphitic shell structure and a large surface area, CNOs possess many excellent physical properties, such as high electrical conductivity and can be used in the fields of magnetic and gas storage materials, lubricants, for nanoreactors or as substrates for catalyst carriers and electrochemical capacitors [183].

## 5. Conclusions and Future Outlook

The conversion of biomass into carbon nanofibers offers the possibility to reduce the high carbon cost and provides an alternative to limited petroleum-based resources. Biomass is an available and sustainable material that can be converted into carbon nanofibers for various applications. Currently, there are few studies on the modification of biomass and the basic knowledge of the natural components of biomass and further conversion into carbon nanofibers, although the application potential is promising. This review of carbon nanofibers produced by electrospinning and their potential applications illustrates their usefulness in various technological and biomedical fields. While carbon nanofibers are produced by an electrospinning technique followed by stabilization and carbonization, their physical and chemical properties make them useful in a variety of applications. However, few studies have been conducted on the application of carbon nanofibers from biomass because this topic is still relatively under-researched. In particular, the available information on the application of biomass-derived carbon nanofibers produced by electrospinning is still limited. The production and optimization of the properties of carbon nanofibers from biomass using different types of biomass provide the opportunity for subsequent scaling up to a commercial scale. This study was aimed to clarify the relevant aspects and contribute to this goal.

This work can serve as a basis for further work on carbon nanofibers from biomass and biomass blends and their applications in energy storage and environmental protection, as well as medical technology and biotechnology in the future. First, there are many works evaluating the production of carbon nanofibers and carbon materials by different methods, but the production of carbon nanofibers from biomass by electrospinning is not yet mature and should be analyzed in-depth using natural materials and biopolymers, which can be an alternative for many environmentally friendly applications. The work also proves the improvement of biomass-derived carbon nanofibers by electrospinning and their application due to their physical and chemical properties. Since this topic is lacking in the literature, this review paper can serve as a basis for further research and facilitate the work of other authors.

Moreover, it is possible to use some additives, such as TiO_2_, Pt, Zr, etc., in the synthesis of CNFs from biomass to increase their surface area and conductivity, which is the key for electrochemical batteries and fuel cells. In conclusion, the use of biomass-derived CNFs from various industrial applications gives a great advantage to some factors, such as cost, performance, stability and the possibility to minimize the environmental damage and to obtain renewable materials as a precursor for the production of CNFs from renewable resources.

## Figures and Tables

**Table 1:** Isothermal treatment conditions of biomass-derived electrospun carbon nanofibers.

Precursors	Heat Treatment	Reference
Polyacrylonitrile (PAN)/gelatine	**Stabilization:** between 240 °C and 300 °C, heating rates between 0.5 K min^−1^ and 4 K min^−1^, 1 h at the final temperature. **Carbonization:** at 800 °C, heating rate of 10 K min^−1^ in N_2_, 1 h at the final temperature.	[14]
PAN/mycelium	**Stabilization:** at 280 °C for 1 h, heating rate of 1 K min^−1^, 1 h at the final temperature. **Carbonization:** at 500 °C for 1 h, heating rate of 10 K min^−1^ in N_2_, 1 h at the final temperature.	[19]
PAN/konjac glucomannan (KGM) Amorphophallus konjac	**Stabilization:** at 280 °C for 1 h, heating rate of 1 K min^−1^, 1 h at the final temperature. **Carbonization:** at 500 °C for 1 h, heating rate of 10 K min^−1^ in N_2_, 1 h at the final temperature.	[111]
Mg(NO_3_)_2_. 6H_2_O/lignin	**Stabilization:** (1) Temperature was increased from 25 to 150 °C, heating rate of 1 K min^−1^, 24 h. (2) Temperature was increased from 150 to 350°, heating rate of 1 K min^−1^, 4 h. **Carbonization:** at 800 °C for 1 h, heating rate of 3 K min^−1^ in N_2_.	[112]
Lignin	**Stabilization:** at 200 °C, heating rate of 0.08 K min^−1^, 48 h at the final temperature. **Carbonization:** at 900 °C, in N_2_, heating rate of 10 K min^−1^, 2 h at the final temperature.	[113]
Cellulose acetate/lignin	**Stabilization:** at 220 °C, heating rate of 0.4 K min^−1^, 12 h at the final temperature. **Carbonization:** 600 °C, heating rate of 4.0 K min^−1^ in N_2_, 2 h at the final temperature.	[72]
H_3_PO_4_/lignin	**Stabilization:** (1) without H_3_PO_4_: at 200 °C, heating rate of 0.08 K min^−1^, 60 h at the final temperature. (2) with H_3_PO_4_: at 200 °C, heating rate of 1 K min^−1^, 1 h at the final temperature. **Carbonization:** at 900 °C, under low concentration of O_2_.	[64]
Lignin/polyvinyl acetate (PVA)	**Stabilization:** (1) Temperature was increased from 25 to 100 °C, heating rate of 10 K min^−1^, 2 h. (2) Temperature was increased from 100 to 180 °C, heating rate of 1 K min^−1^, 16 h. (3) Temperature was increased from 180 to 220 °C, heating rate of 0.5 K min^−1^, 8 h. **Carbonization:** Temperature was increased from 25 to 1200 °C, heating rate of 5 K min^−1^ in argon, 1 h.	[69]
Cellulose/ chitosan	**Stabilization:** at 270 °C, heating rate of 2 K min^−1^, 2.5 h at the final temperature. **Carbonization:** at 900 °C, heating rate 2 K min^−1^, 2 h at the final temperature.	[114]
PVA/ walnut shell powder	**Carbonization in one-step:** between 800 and 1200 °C, heating rate of 5 K min^−1^, 1 h at the final temperature.	[115]
Tar/PAN/ silver (Ag)	**Stabilization:** at 300 °C, heating rate of 1 K min^−1^, 1 h at the final temperature. **Carbonization:** 900 °C, heating rate of 5 K min^−1^ in N_2_, 1 h at the final temperature.	[116]
NFKP/Ni–Co Typha domingensis	**Stabilization:** 12 h at 200 °C. (unspecified heating rate) **Carbonization:** at 700 °C, argon, 3 h (unspecified heating rate)	[117]
Aconitum sinomontanum Nakai/PAN	**Stabilization:** at 280 °C, heating rate 1 K min^−1^, 3 h at the final temperature. **Carbonization:** at 800 °C, heating rate of 2 K min^−1^ in N_2_, 1 h at the final temperature.	[118]
Lignin/PAN; Lignin/PAN/KOH	**Stabilization:** at 220 °C, heating rate 0.5 K min^−1^, 4 h at the final temperature. ****Carbonization:**** (1) At 1000 °C in N_2_, heating rate of 4 K min^−1^, 4 h (2) Lignin/PAN + KOH; 800 °C, heating rate of 4 K min^−1^ in N_2_, 1 h	[119]

## Data Availability

Not applicable.
